# Enhanced photoluminescence stability of CdS nanocrystals through a zinc acetate reagent†

**DOI:** 10.1039/c8ra03504k

**Published:** 2018-07-16

**Authors:** M. Banski, M. Chrzanowski, G. Zatryb, J. Misiewicz, A. Podhorodecki

**Affiliations:** Department of Experimental Physics, Wroclaw University of Science and Technology Wybrzeze Wyspianskiego 27 50-370 Wroclaw Poland mateusz.banski@pwr.edu.pl artur.p.podhorodecki@pwr.edu.pl

## Abstract

In this study, the role of a zinc acetate precursor in improving the luminescence stability of purple-emitting CdS nanocrystals is investigated. The oleate-capped core of CdS nanocrystals exhibits intense photodarkening under prolonged UV excitation. From the results of photoluminescence experiments, we can observe that photobleaching is responsible for the degradation of temporal stability, *i.e.*, decline in photoluminescence intensity. Herein, we demonstrate that by adding zinc acetate to the synthesis solution, one can enhance the photoluminescence stability by the complete suppression of the bleaching processes of nanocrystals. We can distinguish between the effects caused by zinc ions and those caused by acetate ligands. Acetate ligands improve the photoluminescence stability of the core of CdS nanocrystals. However, only when zinc acetate is used, the PL stability can be conserved at high excitation power. Simultaneously, we have studied the influence of zinc cations and acetate ligands on the kinetics of nanocrystal growth. The presented results underline the importance of short surface capping ligands and zinc cations in CdS nanocrystal synthesis. This study exhibits a new advantage of exploiting zinc acetate reagents in one-pot nanocrystal synthesis.

## Abstract 2

An enhancement in the photoluminescence temporal stability was observed upon zinc acetate addition to CdS nanocrystals in one pot synthesis.

## Graphical abstract

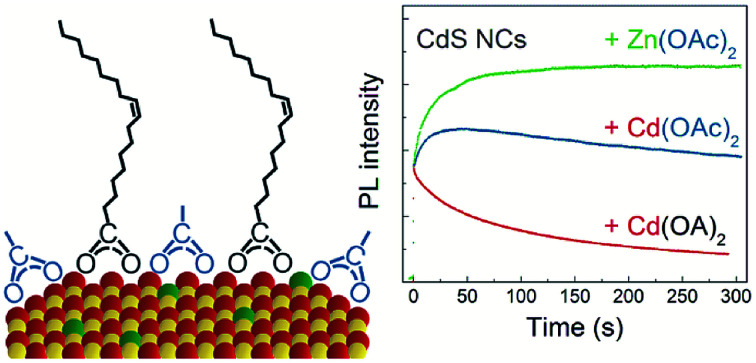

## Introduction

Understanding the impact of synthesis precursors on the structural homogeneity/recurrence and optical properties of colloidal nanocrystals (NCs) is a key issue in the manufacturing of high quality and stable light emitters based on colloidal quantum dots.^1^ Luminophores despite a spectrally narrow emission and high photoluminescence quantum yield also require temporal stability, especially at short wavelengths (blue and purple), for lightning applications;^2^ this is hardly viable in case of bare NC cores, because small NC dimensions result in large amount of defect states at their surface. Surface passivation using semiconducting materials with wide band gaps results in CdS/ZnS core-shells or CdZn_*x*_S_1−*x*_ alloy NCs, and this leads to the improvement in luminescence properties.^3^ These heterostructures exhibit long luminescence lifetimes of trions, leading to high suppression of charging events associated with Auger processes and enhancement in luminescence stability.^4–6^

Recently, acetate-based precursors were shown to have strong impact on many optical and structural properties of nanostructures (nanobelts, nanorods and other structures).^7^ Houtepan *et al.* have reported the influence of lead acetate on the dimensionality and shape of PbSe NCs.^8^ Advanced structures such as CdSe platelets have been obtained using cadmium acetate.^9^ Acetate reagents also alter reaction kinetics, leading to fast rates of nucleation and growth, as reported by Liu and co-workers.^10^

In this article, we present a heating-up synthesis procedure of highly monodisperse CdS NCs with a zinc acetate precursor, which results in the production of CdS:Zn NCs. We have investigated the role of zinc acetate in altering NC growth. Furthermore, we show that by using the zinc acetate reagent, temporal photoluminescence (PL) stability can be improved under an intense laser beam excitation. We have analysed whether zinc ions or acetic acid moieties are responsible for the observed changes in the PL activation (PLA) and degradation (PLD) rates.

## Materials and methods

### Materials

All reagents were purchased from Sigma-Aldrich and used for the synthesis of NCs as received including cadmium oxide CdO (99.5%), cadmium acetate Cd(OAc)_2_ (98%), zinc acetate Zn(OAc)_2_ (99.99%), sulfur (99.5%), oleic acid OA (90%) and 1-octadecene ODE (90%).

### Synthesis of CdS:Zn nanocrystals

The synthesis was carried out by a heating-up method. The samples were prepared at 230 °C in a set, which consisted of three samples prepared using various amounts of Zn(OAc)_2_ reagent: 0, 0.1 and 0.2 mmol.

At first, cadmium oleate Cd(OL)_2_ was prepared. For that purpose, CdO (51.25 mg, 0.4 mmol), OA (0.4 ml, 1.25 mmol) and ODE were placed in a 50 ml three-neck flask, degassed for 10 min at room temperature, and then heated to 250 °C under an N_2_ atmosphere. After 30 min, the colourless solution of Cd(OL)_2_ was cooled to 80 °C, and sulphur (6.55 mg, 0.2 mmol) and an appropriate amount of Zn(OAc)_2_ precursor were added to obtain different Zn/Cd molar ratios: 0/4, 1/4 and 1/2. The reaction mixture was then degassed for 20 min at 90 °C and heated to 230 °C. The reaction was carried out for 1 h. To monitor NC growth, 1 ml aliquots were taken at 1, 2, 4, 6, 9, 15, 30 and 60 min during the synthesis. The reaction time was recorded from 170 °C. Samples were purified with acetone and dispersed in toluene for spectroscopic measurements.

According to the synthesis route described above, a single reaction using 0.2 mmol of Cd(OAc)_2_ instead of Zn(OAc)_2_ was carried out for verification of the influence of acetate ions on PL stability. In another synthesis, an additional amount of 0.2 mmol Cd(OL)_2_ was used instead of Zn(OAc)_2_ to exclude the effect of additional cations in the reaction solution.

### Structural characterization

Transmission electron microscopy (TEM) images were obtained on an FEI Tecnai G^2^ 20 X-TWIN microscope. Samples were prepared by evaporation of diluted solutions of purified NCs on carbon-coated copper grids. The NC composition was determined by energy-dispersive X-ray spectroscopy (EDX). X-ray powder diffraction (XRD) patterns were measured on a Bruker D8 AXE diffractometer (Cu-K_α_). Room temperature micro-Raman scattering was measured using a single-stage spectrometer (T64000 Horiba Jobin Yvon) equipped with a silicon CCD camera. An Ar^+^ laser (*λ* = 514.5 nm) was used as the excitation source. Atomic composition of samples was determined by inductively coupled plasma optical emission spectrometry (ICP-OES) using 8.16 mg CdS:Zn NCs sample, which was diluted in HNO_3_ at 90 °C, dried and dispersed in deionized water.

### Optical characterization

PL spectra were measured at 350 nm excitation wavelength using a Xe lamp coupled with a monochromator. The spectra were obtained on an optical fiber and recorded using a CCD spectrometer (AvaSpec-ULS2048XL). Absorption (ABS) spectra were measured on a JASCO V-570 spectrophotometer.

Time stability series of the emission of NCs were carried out on an AvaSpec-ULS2048XL spectrometer. We used 405 nm laser (CNI laser) of 115 mW total power and grey filter for various power excitations. Samples for the measurements of temporal stability were prepared by spin-coating a diluted solution of NCs on microscopic slides.

Absorbance spectra in the NIR range were measured on a Nicolet iS10 FTIR spectrometer equipped with an attenuated total reflectance (ATR) accessory.

## Results and discussion

### Atomic and ligand composition of the nanocrystals

**Fig. 1:**
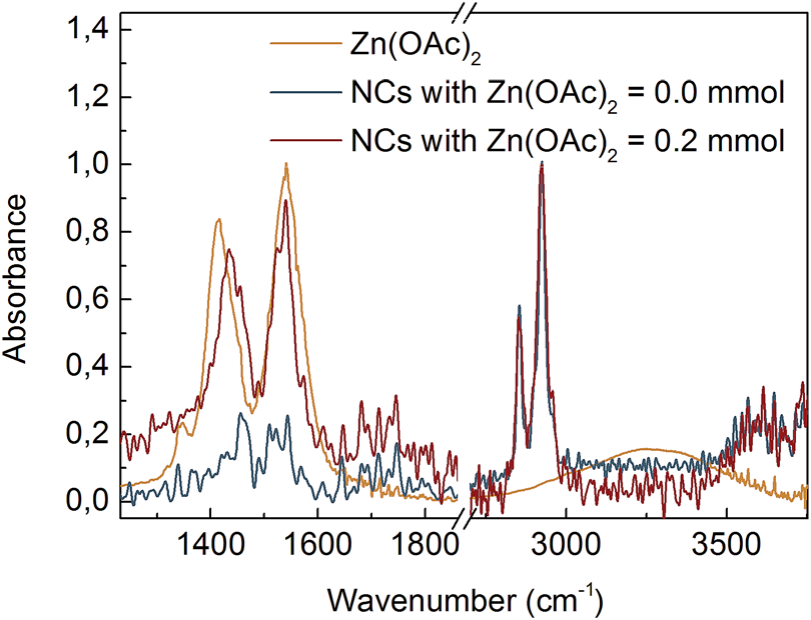
FTIR spectra of Zn(OAc)_2_ precursor and CdS:Zn NCs prepared with various Zn(OAc)_2_ doses. NCs spectra were normalized at 2925 cm^−1^.

The composition of the ligands bound to the NC surface was determined by FTIR spectroscopy. Fig. 1 shows spectra of CdS NCs synthesized with and without 0.2 mmol of Zn(OAc)_2_. Two intense bands centred at 2854 cm^−1^ and 2925 cm^−1^ were due to symmetric and asymmetric –CH_2_– stretching modes, respectively; for our NCs, these bands were assigned to the hydrocarbon chain of the ligands. In all the spectra, there was a lack of an intense peak at ∼1712 cm^−1^ associated with the –COOH group, which means that unbound ligands were efficiently separated from NCs during purification. Instead, two peaks appeared at ∼1542 cm^−1^ and ∼1435 cm^−1^, which could be assigned to the asymmetric (*ν*_as_) and symmetric (*ν*_s_) stretching vibrations of ionized –COO^−^ groups.^11^ An increase in the –COO^−^ peak intensity relative to that of the –CH_2_– bands when the Zn(OAc)_2_ precursor was used in the synthesis suggested that long oleate ligands (CH_3_(CH_2_)_7_CH=CH(CH_2_)_7_COO^−^) were partially substituted by short acetate (CH_3_COO^−^) ligands at the CdS NC surface.

**Fig. 2:**
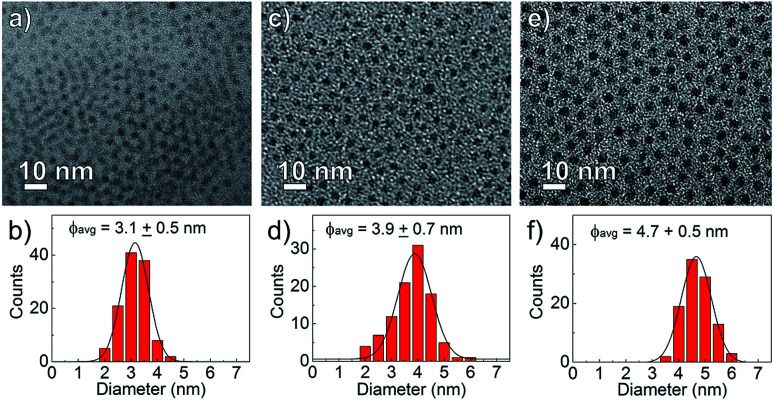
TEM images and NC size distributions of CdS:Zn NCs synthesized at 230 °C with various Zn precursor doses (a and b) Zn(OAc)_2_ = 0 mmol, (c and d) Zn(OAc)_2_ = 0.1 mmol and (e and f) Zn(OAc)_2_ = 0.2 mmol.

The nanocrystal morphology was determined *via* analysis of TEM images. The synthesized CdS:Zn NCs were characterized by a spherical shape and high monodispersity, as confirmed by the TEM images shown in Fig. 2. The particle size distributions were calculated, and an increase in the average NC diameters correlated well with a higher dose of Zn(OAc)_2_, *i.e.*, the mean NC size ranged from 3.1 to 4.7 nm when the amount of Zn precursors increased from 0 to 0.2 mmol; the other synthesis parameters were unchanged.

The main goal of the structural analysis was to determine the internal crystal structure of CdS:Zn NCs, which may be an alloy, gradient or core–shell structure. Recently, W. Zhang *et al.* proposed a similar protocol for the synthesis, concluding a core/gradient alloy internal structure of the obtained CdS/Zn_*x*_Cd_1−*x*_S NCs;^12^ they started the reaction using CdO, OA, Zn(OAc)_2_ and sulphur. The authors concluded that the direct use of CdO as a Cd source and its abrupt activation at elevated temperatures is a key to the successful heterogeneous nucleation and controlled growth of the core/shell NCs. They also mentioned that when activated cadmium stearate was used, Zn_*x*_Cd_1−*x*_S NCs were formed. Thus, based on this above-mentioned study, we may expect an alloy structure for our CdS:Zn NCs.

Fig. S2† shows high resolution (HR) TEM images of the synthesized CdS:Zn NCs; these images show uniform nanoparticle interior, thus do not confirm the presence of a core/shell structure.

EDX experiments were performed to evaluate if zinc cations were incorporated into the synthesized CdS NCs. EDX spectra (Fig. S3†) indicated that a small amount of zinc is present in NCs. The molar ratio of zinc to total cations of CdS NCs as a function of the number of moles of Zn(OAc)_2_ is plotted in Fig. S4.† The experimentally obtained ratios were smaller than those expected based on a calculation assuming proportional contributions of Cd and Zn precursors.

Precise results of the NC composition were obtained from the ICP-OES experiments. When 0.2 mmol of Zn(OAc)_2_ was added during the CdS NC synthesis at 230 °C, 12.3% of NCs cations were found to be Zn. Taking into account the NC diameter (4.7 nm from Fig. 1f) and assuming a core/shell structure, the possible CdS core was found to be 4.4 nm in diameter, and the average thickness of the ZnS shell was around that of one monolayer (ML). One ML is a very thin shell; however, there are reports providing information that 1 ml shell is sufficient to enhance PL intensity.^13^ The elemental analysis confirmed that sufficient Zn cations were incorporated into NCs to form a core/shell structure. However, the spatial distribution of Zn within NCs remained unexplained at this point.

**Fig. 3:**
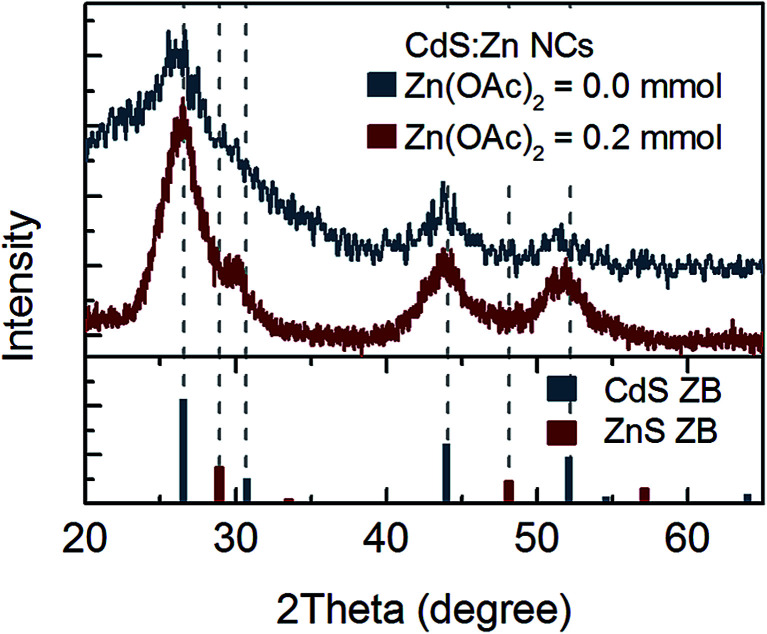
XRD spectrum of CdS:Zn NCs synthesized with 0.2 mmol of Zn(OAc)_2_ and reference peak positions for ZB CdS and ZnS powder.

Thus, further structural analysis was performed. XRD analysis of bare CdS NCs clearly indicated a zinc blend (ZB) crystal structure (Fig. 3). No shift of CdS(ZB) diffraction peaks was observed for NCs synthesised with 0.2 mmol zinc precursor. However, a new peak at ∼30° appeared between the (111) peak of ZnS(ZB) and the (002) peak of the CdS(ZB) structure; its origin was not clear, since it could not be simply assigned to the ZnS shell or Zn_*x*_Cd_1−*x*_S alloy. Nevertheless, taking into account that Zn was truly incorporated into NCs (according to the above-mentioned elemental analysis), this peak may be assigned to the impact of Zn on the crystal structure of CdS:Zn NCs.

**Fig. 4:**
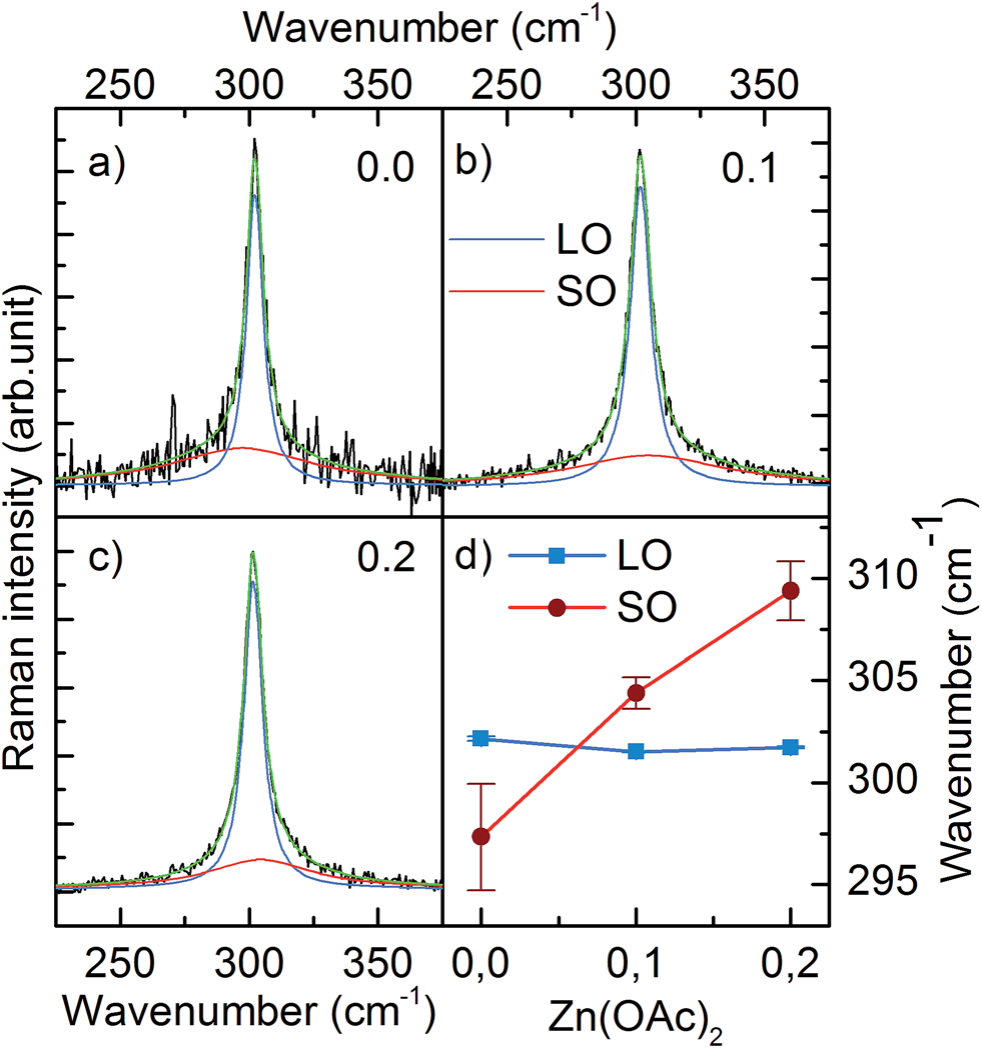
Raman spectra of LO and SO bands of CdS:Zn NCs prepared with various Zn(OAc)_2_ doses (a) 0.0 mmol, (b) 0.1 mmol, (c) 0.2 mmol. (d) Shows the position of LO and SO bands as a function of Zn(OAc)_2_ dose.

We also performed Raman scattering measurements to investigate a possible zinc remnant in CdS NCs. A longitudinal optical phonon (LO) band originating from CdS centered at ∼301.6 cm^−1^ dominated the spectrum. Fitting the spectrum with one Lorentzian function resulted in an *R*^2^ factor equal to ∼0.9. Higher goodness of fit (*R*^2^ = 99) was achieved by adding the second Lorentzian function describing the low intensity band located at shorter wavenumber (Fig. 4a). Based on the results reported in literature, we assigned this band to surface optical phonons (SO).^14^ Moreover, Fig. 4d clearly shows that the SO band shifted to a higher wavenumber when 0.1 and 0.2 mmol of Zn(OAc)_2_ were added to the synthesis. This shift of the SO band followed the wavenumber characteristic for SO modes of ZnS.^15^ Simultaneously, the LO band of CdS remained unchanged. Thus, taking into account the previously discussed TEM, ICP and XRD results, we could conclude that our CdS:Zn NCs crystallized as a CdS core, whereas the admixtured Zn ions formed a thin Zn_*x*_Cd_1−*x*_S alloy shell.

### Optical properties

**Fig. 5:**
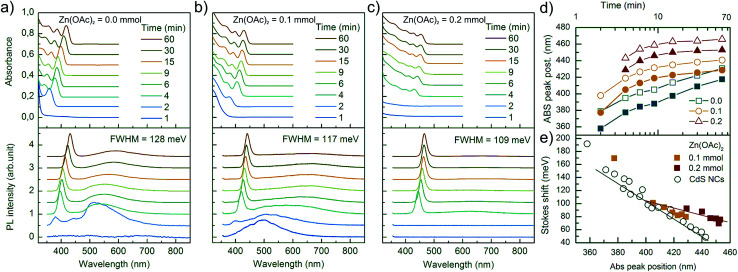
ABS and PL spectra of CdS:Zn NCs synthesized at 230 °C with various Zn(OAc)_2_ precursor doses (a) 0 mmol, (b) 0.1 mmol and (c) 0.2 mmol. (d) Positions of the first exciton transition determined from ABS and PL spectra as a function of synthesis time. (e) Stokes shift as a function of the ABS peak position. Data marked as open circles refer to the three samples of CdS NCs marked in the text as Zn(OAc)_2_ = 0.0 mmol, Cd(OAc)_2_ = 0.2 mmol, and Cd(OA)_2_ = 0.2 mmol.

Optical spectroscopic analysis was performed to observe the tuning of ABS and PL peaks due to NC growth and to determine how Zn(OAc)_2_ added to the synthesis influenced the kinetics of growth of NCs and their optical properties. The PL spectra were measured using a 405 nm laser, which sufficiently excited all samples above the first excitonic transition. The evolution of PL and ABS spectra of NCs during the synthesis is presented in Fig. 5. Three series of presented spectra correspond to the synthesis with various Zn precursor doses: (a) 0 mmol, (b) 0.1 mmol and (c) 0.2 mmol. Part (a) of Fig. 5 refers to the optimized synthesis of CdS NCs; this particular synthesis was characterized by a balanced, slow growth of NCs, which exhibited a mean diameter of 3.1 nm and a narrow size distribution, appearing as narrow Full Width at Half Maximum (FWHM) of the PL peak, and its value was 128 meV (20 nm at 440 nm); this value is typical for CdS NCs.^16,17^

Comparing the three series of samples, one can observe that the increase in Zn(OAc)_2_ precursor dose suppresses the broad emission band located on the long wavelength side of the excitonic emission. This wide band is ascribed to the mid-gap defect state emission.^18^ Therefore, the addition of zinc acetate precursor improves either the internal crystal structure of NCs or their surface passivation by zinc or acetate moiety.

In the ABS spectra of CdS NCs, at least three absorption bands could be well-distinguished (Fig. 5a). When Zn(OAc)_2_ was introduced into the synthesis (Fig. 5b and c), the broadening of the absorption bands increased, and the peak separation decreased; this result was in contrast to the reduction of FWHM of the PL bands, which decreased from 128 to 109 meV for CdS and CdS:Zn (0.2 mmol) NCs, respectively. In the literature, authors have assigned such features to the formation of a core/shell structure due to an epitaxially grown shell.^19^

To verify this hypothesis, we synthesized two CdS NC samples having additional amounts of 0.2 mmol of Cd(OAc)_2_ and Cd(OL)_2_ precursors. The presence of the additional amount of Cd(OL)_2_ resulted in a blue shift of the ABS peak position to 405 nm for the samples synthesized for 60 minutes, which indicated a decrease in the NC diameter as compared to that observed for the synthesis without additional precursor. When Cd(OAc)_2_ was added to the synthesis, NCs grew to a significantly larger size, and the ABS peak reached 442 nm after 60 minutes of the synthesis. The broadening of the absorption bands increased, ABS peak separation decreased, and the absorbance significantly increased at short wavelengths (<350 nm) as compared to the absorption at the first excitonic ABS band. The same changes in the ABS spectra were observed for NCs synthesized with additional Zn(OAc)_2_ precursors. However, while the core/shell structure was possible for CdS NCs synthesized with Zn(OAc)_2_, it was impossible when Cd ions only due to Cd(OAc)_2_ addition were introduced into NC synthesis. Thus, the observed features are thought to result from the acetate ions stabilizing NC surface.

The influence of the acetate reagent on the growth kinetics is clearly visible in Fig. 5d and S4c.† The positions of the first excitonic transition of ABS and the main emission band of PL were plotted *versus* time. The addition of the Zn(OAc)_2_ precursor induced longer nucleation time, a larger initial size of NCs, and a faster rate of NC growth, which resulted in a larger final size of NCs with a wider size distribution. Similar results were observed when Cd(OAc)_2_ was added instead of Zn(OAc)_2_ precursor (Fig. S4c†). Thus, we concluded that acetate ions are the main factor responsible for the changes in NC nucleation and growth kinetics.

The observed phenomenon is consistent with previously reported results concerning other metal acetates. Lead acetate can induce rapid nucleation/growth of PbS NCs.^8^ Partial replacement of oleic acid molecules by lead acetate molecules has been proved to result in large NCs due to weak forces between short acetic molecules and easier nanocrystal attachments.^10^

The effects of Zn on the kinetics of NC growth are more subtle. CdS:Zn (0.2 mmol) NCs reached their final diameter after 15 minutes and not after 30 minutes, as CdS NCs with 0.2 mmol of Cd(OAc)_2_ did (Fig. S4c†). In Fig. S4a,† the PL spectra show that Cd(OAc)_2_ did not suppress the defect state emission as Zn(OAc)_2_ did (Fig. 5c). The other important influence of Zn^2+^ ions on CdS:Zn NCs is the increase in Stokes shift. Fig. 5e shows the Stokes shift for CdS NCs obtained from three different syntheses (open black circle), and the results were compared with the results of Zn-doped CdS NCs doped with 0.1 and 0.2 mmol Zn(OAc)_2_ precursor doses (filled squares). The large value of the observed Stokes shift for small CdS NCs was in agreement with the predictions of Yu *et al.*,^20^ and it monotonically decreased with the decreasing energy gap, *i.e.*, with the increasing diameter of CdS NCs. For CdS:Zn NCs, the Stokes shift did not follow the expected trend, *i.e.*, the Stokes shift was larger for CdS:Zn NCs as compared to that for CdS NCs, and the difference increased with the diameter of NCs, which is especially significant for a high Zn dose. This is an important result confirming that Zn contributing to CdS NCs influences their band structure.

In general, a difference in the lattice constants of the core and shell causes strain.^21^ For example, Phadnis *et al.* have shown that the growth of 4 monolayers of ZnS shells at the CdS core reduces the Stokes shift due to a strain-induced energy level modification from 36 to 5 meV.^22^ However, in our case, a reverse trend is observed, *i.e.*, an increase in the Stokes shift when Zn(OAc)_2_ is added to the synthesis. Thus, instead of a core/shell structure, Zn ions may form an alloy with the CdS core and introduce new energy levels to the energy structure of NCs characterized by different oscillation strengths.^23^ Thus, the contributions of particular energy levels to the absorption and emission spectra can be different, giving rise to the observed large Stokes shift.^24^

### Photoluminescence stability

From PL spectra, we concluded that Zn restricts low energy defect state photoluminescence. To further determine the influence of Zn(OAc)_2_ on the photoluminescence properties of the synthesized CdS:Zn NCs, temporal PL stability measurements were conducted. Thin NC films were illuminated for 5 min under ambient conditions to verify temporal stability of the PL intensity.

Changes in PL intensities measured for a group of colloidal quantum dots have been previously modelled by several groups and explained by fluorescence intermittency in single quantum dots.^25^ Especially, quantum dot charging and Auger processes were suspected to be responsible for the observed quantum dot blinking. The on/off statistics, observed for a single quantum dot, leads to the overall temporal behaviour of photoluminescence of the quantum dots ensemble. For example, a consecutive elementary reaction (CER) scheme, written as A → B → C, was used to model the PL activation and degradation (where A is the fractions of quantum dots in the dark, non-emissive (charged) states, B – in emissive bright states, and C – fully inactive, photobleached states).^26^ Cordero *et al.* proposed that PL degradation arises from the formation of surface traps due to ligand desorption or photooxidation.^27^ Thus, the time traces have been found to be both wavelength- and excitation power-dependent.^26,28^

**Fig. 6:**
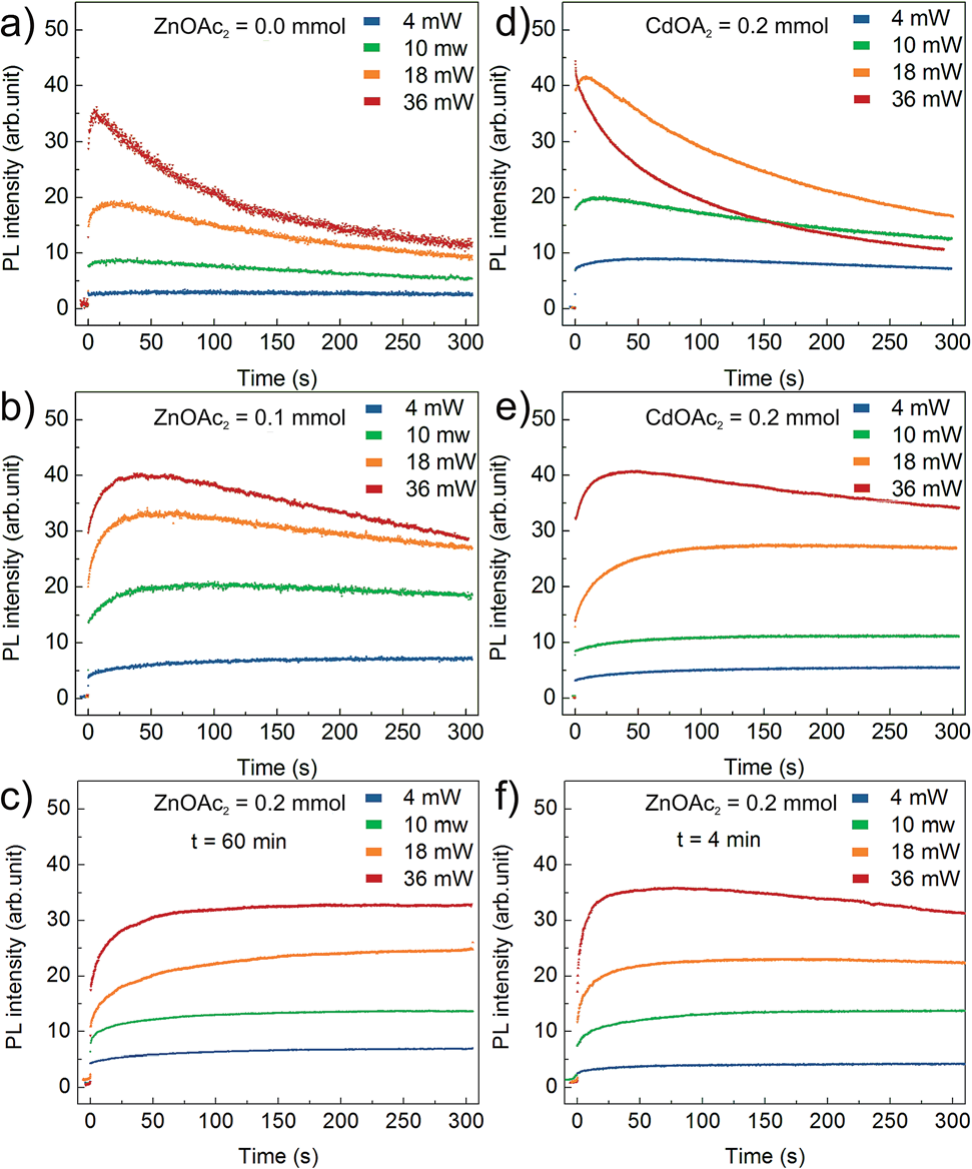
PL intensity time traces of CdS:Zn NCs under 405 nm illumination. Results in (a)–(c) refer to NCs synthesized at 230 °C for 60 minutes with increasing Zn-precursor dose. Reference experiments conducted on samples synthesized with (d) additional amount of Cd(OL)_2_, (e) additional amount of Cd(OAc)_2_ and (f) CdS:Zn NCs synthesized with Zn(OAc)_2_ = 0.2 mmol for 4 minutes only.

Fig. 6 presents the results of the PL temporal stability for NCs synthesised with varying Zn(OAc)_2_ precursor doses: (a) 0 mmol, (b) 0.1 mmol and (c) 0.2 mmol. In general, the recorded time traces are characterized by an initial fast rise of the PL intensity followed by its slow decay. The observed PL rise and decay may be assigned to PL activation (PLA) and degradation (PLD), respectively.^25^ The higher the excitation power, the faster the PLA and PLD rates; this is clearly observed for bare CdS NCs presented in Fig. 6a. The PLD rate is even faster for CdS NCs prepared with an additional amount of Cd(OL)_2_ precursor (Fig. 6d). The effect of the precursor composition is clearly visible in Fig. 6b and c, where a significant decrease in the PLD rate is observed with an increase in the Zn(OAc)_2_ precursor dose. The time constant of PLD is at least one order of magnitude larger for CdS:Zn (0.1 mmol) NCs (Fig. 6b) compared to that for bare CdS NCs (Fig. 6a). At higher Zn concentration (0.2 mmol), the PL degradation of CdS:Zn NCs is completely suppressed, and no decay in the PL intensity is observed (in Fig. 6c) within the time of the experiment (5 minutes). Additionally, from Fig. 6f, it can be seen that small CdS:Zn (0.2 mmol) NCs (the sample taken after 4 minutes of synthesis) exhibit a limited PLD only at the highest excitation power. This experiment confirms that better temporal stability of the PL intensity arises from Zn and/or OAc directly and not simply due to the large size of NCs obtained when Zn(OAc) is added to the synthesis. Moreover, this result confirms that better temporal stability of PL is present from the beginning of the synthesis. The inhibition of PLD in case of CdS:Zn NCs confirms the efficient passivation of the defect states for bare CdS NCs. This result is consistent with the above-discussed result of the reduction of the defect state emission originally observed in the PL spectra for bare CdS NCs, which reduces to a minimum for CdS:Zn NCs (Fig. 4).

To improve our understanding of the role of Zn(OAc)_2_ in improving PL stability, additional experiments were performed on CdS NCs synthesized with an adequate molar amount of Cd(OAc)_2_ instead of Zn(OAc)_2_. In that way, we distinguished the influence of acetate ions and zinc cations. The obtained results indicated that acetate ions improved PL stability, as presented in Fig. 6e. However, at high excitation power, NCs synthesized with Zn(OAc)_2_ did not show any PLD, whereas those synthesized with Cd(OAc)_2_ did. Thus, both Zn and acetate ions have crucial influence on the PL temporal stability.

## Conclusion

In conclusion, we show that the zinc acetate precursor used in one-pot synthesis enables the control of the reaction kinetics as well as the final size of CdS NCs. The synthesised NCs are monodispersed, containing up to 12% of zinc, and they are stabilized by both acetate and oleate ligands. Furthermore, we observe that the addition of Zn(OAc)_2_ supresses the defect state emission, resulting in pure excitonic emission, which provides great resistance to the degradation process under UV illumination in broad ranges of excitation powers. The conducted experiments confirm that both Zn and acetate ions modify the optical properties of NCs, which is observed by the increase in the Stokes shift, and both are needed for increasing the PL temporal stability. The most probable form of NCs is a CdS core with Zn_*x*_Cd_1−*x*_S alloy shell, whereas acetate is a co-ligand on the NC surface.

## Conflicts of interest

There are no conflicts to declare.

## Supplementary Material

RA-008-C8RA03504K-s001
