# Supplementary material for: Enhanced photoluminescence stability of CdS nanocrystals through a zinc acetate reagent
Source: RSC Adv. 2018 Jul 16;8(45):25417–22. doi: 10.1039/c8ra03504k (PMC9082552; doi:10.1039/c8ra03504k)
Supplement: RA-008-C8RA03504K-s001 [file RA-008-C8RA03504K-s001.pdf]

Electronic Supplementary Material (ESI) for RSC Advances.
This journal is © The Royal Society of Chemistry 2018

Enhanced photoluminescence stability of CdS nanocrystals through a zinc acetate reagent†

M. Banski,[a] M. Chrzanowski,[a] G. Zatryb,[a] J. Misiewicz,[a] A. Podhorodecki,[a]

[a] Department of Experimental Physics, Wroclaw University of Science and Technology, Wybrzeze Wyspianskiego 27, 50-370 Wroclaw, Poland.

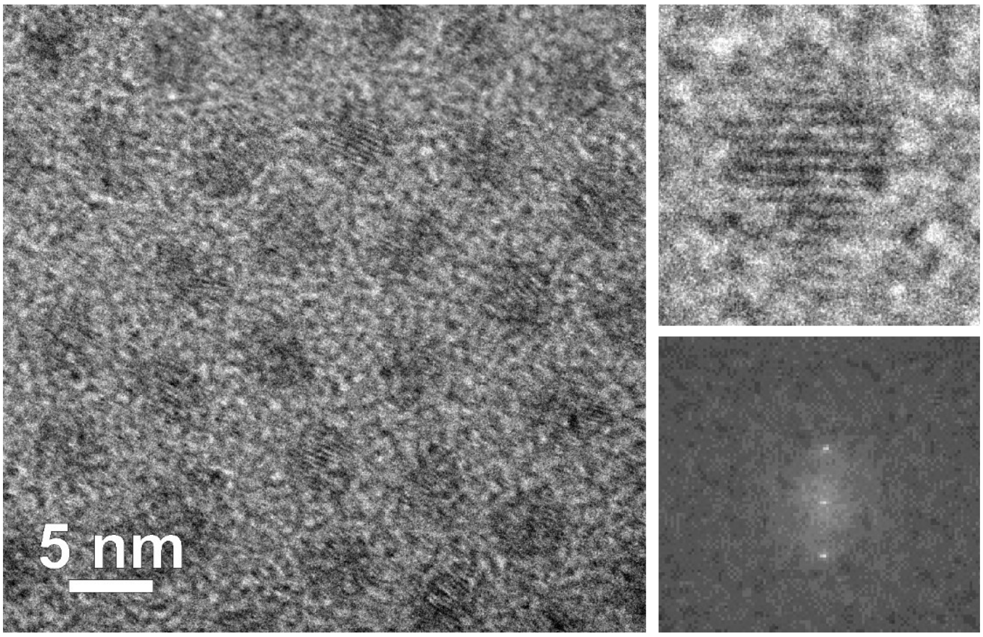

Fig. S1. HR-TEM images and electron diffraction of CdS:Zn NCs (0.2 mmol of Zn precursor).

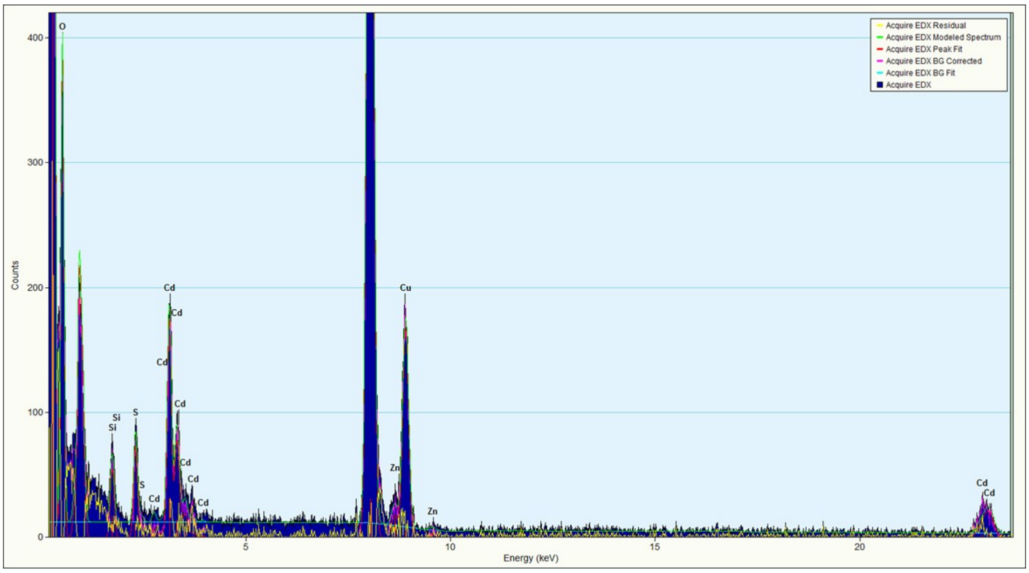

Fig. S2. EDX of CdS:Zn synthesized with 0.2 mmol of $Zn(OAc)_2$.

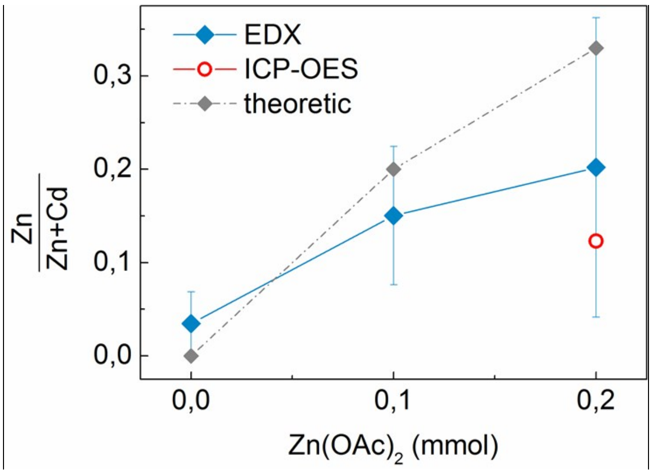

Fig. S3. Molar ratio of zinc to total cations of CdS:Zn nanocrystals determined from EDX spectra and ICP-OES in a function of $Zn(OAc)_2$ dose.

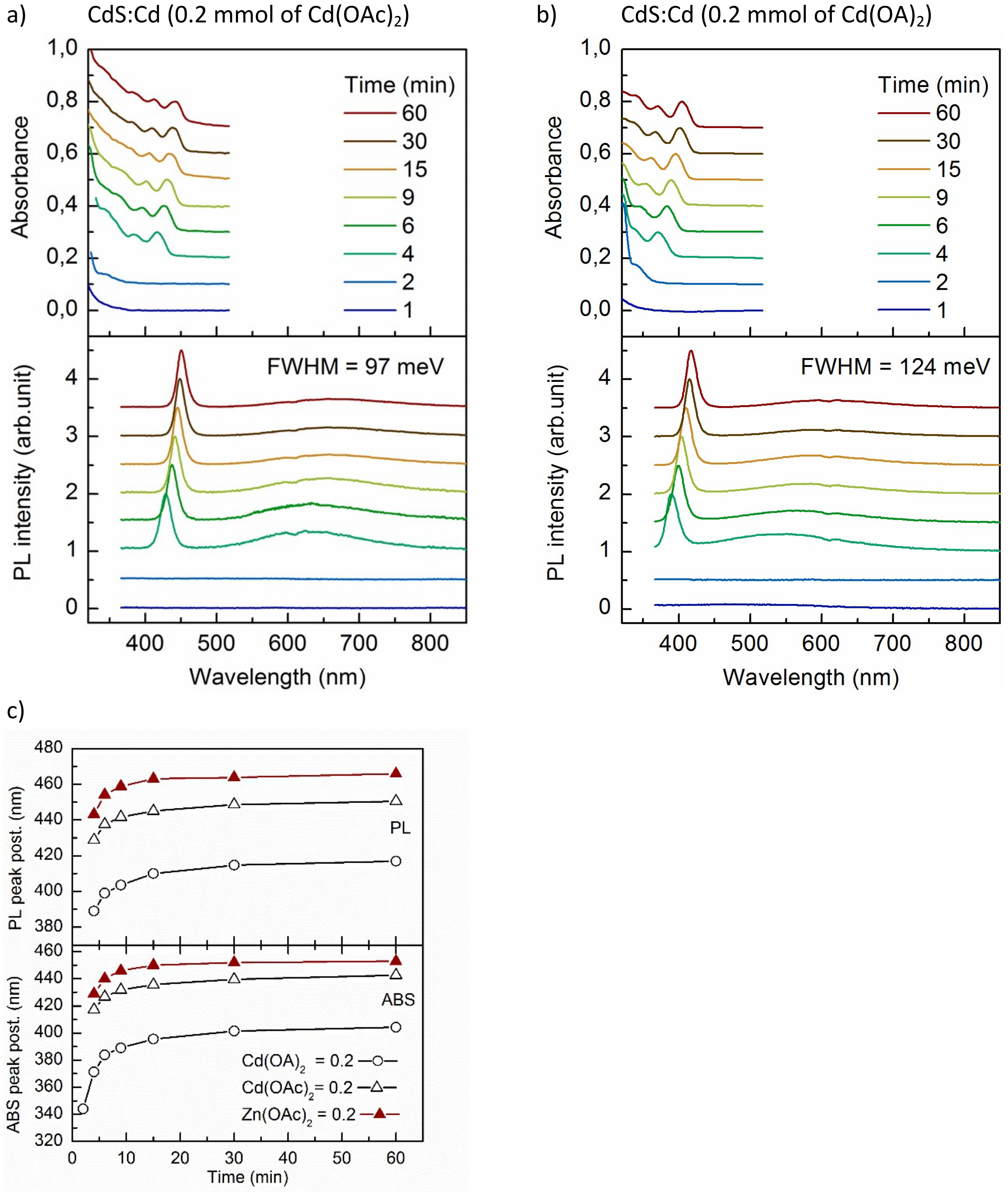

Fig. S4. ABS and PL spectra of CdS NCs synthesized with (a) 0.2 mmol $Cd(OAc)_2$ and (b) 0.2 mmol $Cd(OA)_2$. (c) Positions of the first exciton transition of above samples determined from ABS and PL spectra in a function of synthesis time compared to results of CdS NCs with 0.2 mmol $Zn(OAc)_2$ addition.

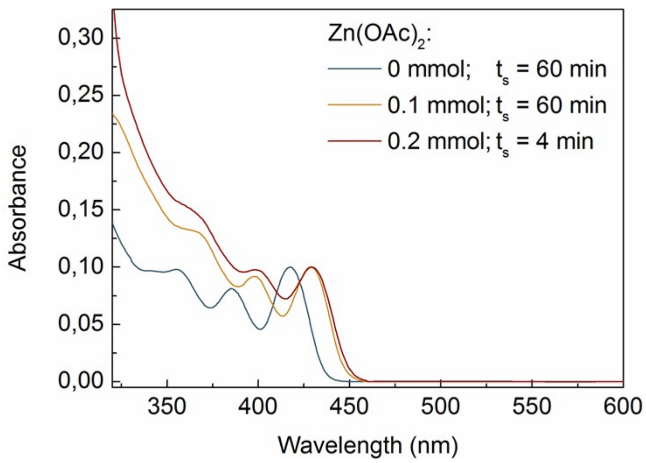

Fig. S5. ABS spectra of selected NCs samples having similar size. A decrease of ABS bands separation due to increase of $Zn(OAc)_2$ concentration is visible.
